# Women’s experiences throughout the birthing process in health facilities in Arab countries: a systematic review

**DOI:** 10.1186/s12978-022-01377-y

**Published:** 2022-03-18

**Authors:** Arein Awad, Aisha Shalash, Niveen M. E. Abu-Rmeileh

**Affiliations:** 1 Institute of Community and Public Health, Birzeit University, P.O.Box 14, Birzeit, Palestine; 2 School of Medicine, University of Limerick, Limerick, Ireland

**Keywords:** Mistreatment of women, Facility-based childbirth, Proxy, Arab countries, Evidence-based typology

## Abstract

**Background:**

Mistreatment of women during facility-based childbirth has become a significant public health issue globally and is gaining worldwide attention. This systematic review of quantitative studies aimed to estimate the prevalence of mistreatment women may experience throughout the birthing process in health facilities in Arab countries. The review also aimed to identify the types of mistreatment, terminology, tools, and methods used to address this topic.

**Methodology:**

The search was conducted using three electronic databases: “PubMed,” “Embase,” and “CINAHL” in May 2020. Studies meeting the inclusion criteria were included and assessed for risk of bias. The analysis was conducted based on the evidence-based typology developed by Bohren et al. as a guide to try to estimate the prevalence of mistreatment.

**Results:**

Eleven studies out of 174 were included. The included studies belonged to only seven Arab countries out of 22 Arab countries. The mistreatment of women during childbirth is still new in the region. Searching within the included studies yielded diverse and indirect terms that were a proxy for the word mistreatment. These terms were not comprehensive to cover different aspects of the topic. The tools that were used to measure the terms widely varied.. Moreover, it was not possible to estimate the prevalence of mistreatment of women due to high heterogeneity among the 11 studies.

**Conclusion:**

The topic of mistreatment of women in Arab countries was not adequately addressed in the studies included in this review. More research on this topic is recommended due to its importance in improving maternal health in the region. However, a standardized and comprehensive terminology for mistreatment of women, a standardized tool, and a standardized methodology are recommended to enable comparability between results and allow pooling to estimate the prevalence.

**Supplementary Information:**

The online version contains supplementary material available at 10.1186/s12978-022-01377-y.

## Plain Language Summary

Childbirth is a highly personal and central event in every mother’s life. However, several studies have documented unpleasant behaviors that women may face throughout the birthing process in health facilities. These behaviors may affect the whole birthing experience, affecting the women’s decision to use health facilities for childbirth in future pregnancies. Therefore, the mistreatment of women during facility-based delivery has become a genuine and global public health issue. There is limited data on this issue in Arab countries. This which triggered the necessity of conducting a systematic review that aimed to estimate the prevalence of mistreatment of women and better understand the burden of this issue regionally, thus reflecting the image on how it might be in Palestine. The search was conducted using three databases and was limited to studies published in English and Arabic, with no restrictions on the publication year. It included all observational studies that reported the prevalence of women's mistreatment throughout the birthing process in Arab countries. Eleven studies were included in this review, and the risk of bias assessed. All were cross-sectional studies. They were done in seven countries out of the 22 Arab countries. It was not possible to estimate the prevalence of mistreatment because the terms, tools, and methodological characteristics varied widely. Further research on the mistreatment of women during childbirth in Arab countries using standardized terminology, tool and methodology is recommended.

**Supplementary Information:**

The online version contains supplementary material available at 10.1186/s12978-022-01377-y.

## Background

Increasing the rate of facility-based childbirth is one of the main pillars in reducing global maternal and neonatal morbidity and mortality [1]. However, many women across the globe experience mistreatment during facility-based childbirth [2–9]. Mistreatment is used as an indicator of the quality of care and affects the mother’s decision to seek and utilize maternal health services for childbirth [10]. Research recently has grown on this global and significant public health issue.

There is a lack of a standardized definition and methodology that addresses mistreatment. Several publications have proposed definitions and conceptual frameworks for understanding the concept [11–13]. In 2015, The World Health Organization (WHO) researchers; Bohren and colleagues presented a new phrase, “mistreatment of women during facility-based childbirth.” They conducted an extensive mixed-method systematic review to develop a standardized typology of what constitutes mistreatment during childbirth using 65 studies from 34 countries [13].

The WHO team identified seven typologies of mistreatment of women during childbirth: “physical abuse, verbal abuse, sexual abuse, stigma and discrimination, failure to meet professional standards of care, poor rapport between women and providers, and health system conditions and constraints” [13].

With the growing recognition of mistreatment of women during facility-based childbirth, the WHO saw a clear need to develop standardized, evidence-based measurement tools that can be applied at a global level to define, measure, and prevent mistreatment [14]. As a result, the WHO initiated a multi-country research study to develop and validate two tools (labor observation and community survey) to measure the mistreatment of women during childbirth in health facilities in four countries: Ghana, Guinea, Myanmar, and Nigeria [14]. It was a two-phased, mixed-methods study, with phase one aiming to develop and validate the two tools that would provide data on the burden of mistreatment comparable across settings and over time [14]. Phase two aimed to apply these tools and report the prevalence of mistreatment in the four countries [15].

Mistreatment of women throughout the birthing process is associated with several factors [16]. These factors include age, number of previous births, attending antenatal care, time of delivery (day or night), method of delivery (vaginal vs. cesarean), marital status, facility sector (governmental or private), education level, economic situation, type and sex of birth attendant during childbirth, birth companion, race, ethnicity, and immigration status [2, 5, 15, 17–22].

The exposure of women to mistreatment during childbirth can lead to severe and adverse health outcomes for the mother or baby [23–25]. These may include feelings of fear, sorrow, disrespect, insecurity for mothers and the expected baby, distrust and lack of confidence in the health care providers, a sense of weakness and powerlessness, and postpartum depression [23, 26, 27].

However, women are expected to encounter skilled health workers who are competent, respectful, and caring. In addition, they desired to receive timely information, effective communication, and physical and emotional support. Women emphasized that all the above expectations and desires are needed for positive health outcomes during facility-based childbirth [28, 29].

Although this problem is faced by women worldwide, there is limited data on their mistreatment during facility-based childbirth in Arab countries. Furthermore, it is unknown what terminologies, typologies, and methods are used to address this topic in this part of the world.

### Review objectives

This systematic review aimed to identify the status of the conducted studies concerning mistreatment, along with the terminology, typologies, and methods used. As well as to try and estimate the prevalence of mistreatment of women during facility-based childbirth. This would provide an evidence-based strategy to alleviate this problem in Arab countries, improve women’s childbirth experiences in health facilities, and reduce overall maternal mortality regionally.

### Research methodology

This systematic review was conducted following a protocol registered on PROSPERO with ID number CRD42020182806 [30].

### Search strategy

The search was conducted using the accessible databases: PubMed, Embase, and CINAHL. The search was done in May 2020 and was limited to studies published in English and Arabic, with no restrictions on the publication year. The search included observational studies that reported the prevalence of women's mistreatment throughout the birthing process in Arab countries (i.e. cross-sectional and cohort). After doing a literature search [4, 10, 13, 31–41] to ensure that terms were not missed; keywords were determined and can be found in Additional file 1.

### Study selection and inclusion criteria

The inclusion criteria included studies that satisfied the following:

1. Women of reproductive age [15–49], giving or giving birth in a health facility, experienced mistreatment during childbirth.
2. Arab Countries (Jordan, Palestine, Syria, Lebanon, Morocco, Mauritania, Algeria, Tunisia, Libya, Sudan, Somalia, Egypt, Saudi Arabia, Yemen, Oman, Qatar, Bahrain, Kuwait, the Comoros Islands, Iraq, Djibouti, and the United Arab Emirates),
3. Cohort or cross-sectional study designs
4. Articles containing experiences of mistreatment reported by women or observed by trained professionals during labor and delivery in a health facility from health workers
5. Articles related to improving birth outcomes, effective communication between women and healthcare providers, positive/negative birth experience, or birth care.

Two reviewers independently screened the studies’ titles and abstracts for inclusion. The full-text screening was also done by two reviewers independently. Both screenings were done using Covidence portal for reviews. Database searches were downloaded to Endnote and then uploads to Covidence. Conflicts during the title and abstract and full-text screenings were resolved through consensus. If an agreement was not reached, a third reviewer was consulted who made the final decision. The reference lists of the included studies were then hand-searched for any other relevant studies. Articles found by reference list were screened in the same way as the other articles.

### Data extraction

The data extraction sheet was built, piloted, and agreed upon by the research team.

The extracted information included the names of the authors, journals, the publication year, the study title, the aim of the study, the study design, country, inclusion/exclusion criteria, sample size, method of data collection, the primary outcomes of each study, outcome measurements, and the results (i.e. prevalence, mean score, number of women reporting the outcome of interest). The data extraction was also conducted by two reviewers independently, and conflicts were resolved by consensus.

### Risk of bias (quality) assessment

The risk of bias was completed by two reviewers independently using the 10-item tool developed by Hoy et al. to assess the risk of bias in prevalence studies [42]. This tool was user-friendly and demonstrated high interrater agreement. It included assessment for the following domains: selection bias, non-response bias, measurement bias, and bias related to analysis [42]. Disagreements were resolved between the two reviewers through consensus.

### Data synthesis

A flow chart was used to show the number of studies remaining at each stage of the selection process. A narrative and descriptive summary tables of the findings were provided for the included studies structured around the articles’ general data, the terminology, the measurement tools, and the types used. The analysis was conducted based on the evidence-based typology developed by Bohren et al. [13] as a guide to try to estimate the prevalence of mistreatment of women throughout the birthing process in health facilities in Arab countries. This evidence-based typology was used because the authors proposed an inclusive definition for mistreatment. They defined a broad scope of categories that consider different sources of mistreatment that may stem from both intentional or unintentional actions either by healthcare providers or from conditions within the health facilities and system [13]. The prevalence was calculated as the average prevalence of all the items included within the outcome of interest.

## Results

### Search findings

The database and hand search of reference lists resulted in 183 studies with nine duplicate studies, giving 174 studies. The title and abstract screening yielded 32 studies that were eligible for full-text extraction, resulting in 11 included studies [43–53]. The list of full text excluded with reasons is presented in Additional file 2.

**Fig. 1:**
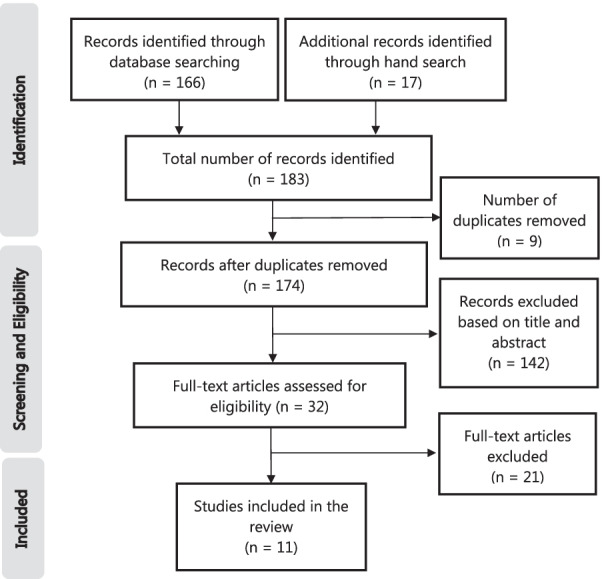
Flowchart of study selection

Figure 1 presents the flow chart of the included studies.

### Characteristics of the included studies

More than half of the studies were conducted between 2010 and 2019. Six out of the 11 studies were published between 2005 and 2014, and the remaining five were published between 2015 and 2020. The majority of the 11 studies were published in journals specific to reproductive health, obstetrics and gynecology, birth, and nursing. All studies were cross-sectional studies. The 11 studies were conducted in seven out of the 22 Arab countries. These countries were Egypt, Jordan, Iraq, Lebanon, Morocco, Syria, and Yemen. The majority of the 11 studies were conducted in health facilities. Specifically, four were conducted in hospitals, two were in clinics, and one was in both settings. The rest were conducted in the community. The data collection method for ten of the 11 studies was an interviewer-administered questionnaire by trained fieldworkers, and one was a self-administered questionnaire and limited to literate women. Sample sizes were relatively large, with the smallest number of participants being 250 women.

The majority of the 11 studies had aims directly related to mistreatment. However, the aim of three of the studies was indirectly related: women’s preferred location of childbirth in case of future pregnancies, trends in postpartum care, and women’s preferences for the type of birth attendant and place of delivery. The characteristics of the included studies are shown in Table 1.

**Table 1:** Characteristics of the 11 Studies

Author	Publication year	Journal name	Country	Setting	Year studied	Method of data collection	Study population	Number of participants	Study aim	Response rate
Ahmed	2020	Reproductive Health	Iraq	Facility-based	2019	Interviewer-administered questionnaire	Women having at least one birth experience (a vaginal delivery) within the past year, either in Rezgary hospital, a Maternity Teaching Hospital or Malafandy, a primary health center in Erbil City	1196	To assess women's perception about the quality of rapport between them and their providers in the delivery room, the association between this satisfaction and sociodemographic characteristics of the women, and the association between women's satisfaction and their general satisfaction with care during labor and delivery	97%
Bashour et al.	2005	BIRTH	Syria	Community/ Household Survey	2003	Interviewer-administered questionnaire	Women of recent delivery of a healthy baby who was less than 3 months old	500	To describe Syrian women's preferences for the type of birth attendant and place of delivery	Not Reported
Boukhalfa et al.	2016	Tropical Medicine and International Health	Morocco	Facility-based	2012	Interviewer-administered questionnaire	Women who had an uncomplicated delivery, a complicated delivery with caesarean section, or complicated delivery without caesarean section when they were about to leave the health facility	973	To assess the financial barriers still faced by households since the FCDP was implemented and hence to understand the degree of its effectiveness	99%
Fort	2012	Reproductive Health Matters	Egypt and Bangladesh	Community/ Household Survey	For Egypt 2005 and 2008	Interviewer-administered questionnaire	Women whose most recent live birth in the 5 years prior to the survey date and were between 15–49 years old	In 2005 was 9845 and in 2008 was 7896	To analyze levels and trends in post-partum care and post-natal care by place of delivery using Demographic and Health Survey data for Egypt in 2005 and 2008 and Bangladesh in 2004 and 2007	Not Reported
Kabakian-Khasholian et al.	2017	Reproductive Health Matters	Egypt, Lebanon and Syria	Facility-based	November 2014 to July 2015	Interviewer-administered questionnaire	Women who gave birth in the postpartum ward before their discharge from the hospital and were18 year**s** old and more	2620	To describe the levels of satisfaction with the childbirth experience and perceptions of control of women giving birth in public hospitals in three middle-income Arab countries, Egypt, Lebanon and Syria, and to determine the service delivery factors associated with their satisfaction	98.6%
Kempe et al.	2010	Sexual & Reproductive Healthcare	Yemen	Community/ Household Survey	Not Reported	Interviewer-administered questionnaire	Women with childbirth experience	220	To examine women's authority at birth with reference to the intrapartum factors, the level of training of staff and the social and demographic background of women	100%
Kempe et al.	2011	ISRN Obstetrics and Gynecology	Yemen	Community/ Household Survey	Not Reported	Interviewer-administered questionnaire	Women with childbirth experience	220	To examine the influence of socio-demographic, delivery outcome, and demand factors on women's preferred location of childbirth in case of a future pregnancy, seen against the background of the previous childbirth	100%
Mohammad et al.	2014	International Nursing Review	Jordan	Facility-based	2012	Interviewer-administered questionnaire	Women who were 7 weeks or more postpartum and had a live term baby	320	To investigate the prevalence and associated factors of dissatisfaction with intrapartum care by Jordanian Women	83.2%
Monazea et al.	2015	Journal of the Egyptian Public Health Association	Egypt	Facility-based	2012	Interviewer-administered questionnaire	All women who had given birth to a single live birth from 4th Jan to 28th Feb 2012 at the public section only of the hospital at the time of discharge	435	To assess women's satisfaction with quality of healthcare during hospitalized delivery and to determine factors associated with their satisfaction	100%
Oweis	2009	International Journal of Nursing Practice	Jordan	Facility-based	2005	Self-administered questionnaire	Women who are literate, have a healthy newborn baby, by normal vaginal delivery and assisted delivery such as forceps and vacuum	177	To document women's perceptions of the different aspects their childbirth experience including expectations, satisfaction and self-control	Not Reported
Sayed et al.	2018	International Journal of Reproduction, Contraception, Obstetrics and Gynecology	Egypt	Facility-based	2016	Interviewer-administered questionnaire	Women who delivered and were at postpartum wards	400	To determine maternal satisfaction towards delivery services at Women's Health Hospital, Assiut University, Upper Egypt	100%

The women included in each study varied in terms of time and place of the interview. Participants were grouped into three main categories based on measuring the outcomes. These were women in postpartum wards before they left the hospital; second, women who had a healthy newborn up to 1 year after delivery in clinics; and third, women with childbirth experience without specifying the period.

### Findings for the terminology and tools used in measuring mistreatment

There are currently multiple phrases being used to express mistreatment, such as “disrespectful care,” “disrespect and abuse,” and “obstetric violence.” None of the included studies yielded any of the terms. Indirect terms were used to reflect mistreatment. These terms fall under the following main categories: women’s satisfaction, perception of control/authority during childbirth, postpartum care, mothers’ experiences of care related to client-provider interaction, mother-infant proximity, other terminologies, and irrelevant terminologies.

*Women’s satisfaction* was reported as the primary outcome in six studies [43, 47, 50–53] and three as the secondary outcome [43, 44, 53]. Satisfaction was measured differently in each of these six studies. For example, “women’s satisfaction”, “dissatisfaction with intrapartum care,” “satisfaction with childbirth experience,” “overall maternal satisfaction with delivery services,” “mother’s satisfaction with delivery care,” and “overall satisfaction of women with the communication of midwives and physicians during labor and birth.” In addition, each of these studies operationalized the satisfaction definition differently. Consequently, each study used a different measurement tool for estimating “satisfaction.”

*Women’s Perception of Control/Authority during Childbirth* was used in four of the 11 studies as the primary outcome [47–49, 51]. These outcomes included “perceived control during childbirth experience,” “women’s perception of control,” “women’s perceived authority at birth,” and “women’s authority during childbirth.” The authors also did not utilize a clear conceptual framework for this term; however, they determined the operational definition differently in each of these studies.

*Postpartum Care* was used in one of the 11 studies [46]. The author used a clarified conceptual definition for the term “postpartum care” and operationalized it using the “demographic health survey (DHS)” that was modified to include information on postpartum and post-natal care for women delivering in health facilities.

One study measured mothers’ experiences of care related to client-provider interaction [53], and another measured mother-infant proximity [49], and both were parts of the questionnaire used in this study.

Other terminologies describe mistreatment used and measured in the studies that could not be classified in the different terms above. These were “privacy sensation during hospital stay” [52], “own choice of birth attendance” [49], “presence of birth support/companion” [49, 53], “not talked to any health professional about how they felt about what happened during labor and birth,” and “attendance of anyone that they did not want to be there” [50]. There were no specific indications for measuring these outcomes since they were extracted from tables within the articles.

There was diversity in the terms and measurement tools used. Each term has an operational definition, making the comparison between these articles difficult.

Considerable variation was observed among the tools used to measure mistreatment. Four studies reported developing their own tools; three were tested/piloted [44, 51, 53], and one was not tested/piloted [43]. Four studies were translated to Arabic [46, 47, 46–47], one study reported only adaptation [52], one study reported only testing[48], and one study adapted and tested/piloted a tool used in an Arab country [45].

For this review—despite having only three of the 11 studies reporting on validation [46, 50, 51]; it was considered that both adaption with testing/piloting or development with testing/piloting, as validation as well.

### Findings for the types identified in measuring mistreatment

The evidence-based typology for mistreatment [13] was used to guide the analysis of this paper. The findings of the 11 studies were mainly classified within two typologies “poor rapport between women and providers” and “health system conditions and constraints.” Table 2 presents typologies with detailed information. Some outcomes were classified under more than one typology; for example, "women’s satisfaction” and “satisfaction with childbirth experience” could be classified under both the sixth typology and the seventh typology, because the scales used to measure both of these outcomes cover several dimensions. Also, Table 2 shows that “satisfaction with childbirth experience” was classified under the fifth category and the sixth and seventh categories because it has one item directly related to the physical examinations and procedures sub-category.

**Table 2:** Summary findings for the types identified, the findings of the studies and the overall range of prevalence

WHO typology	Outcome of tnterest	Findings	Reported/calculated prevalence	Overall range of prevalence
Typology No. Six: “Poor rapport between women and providers”				
“Ineffective communication”	“Overall satisfaction of women with communication of midwives and physicians during labor and birth” [47]	58.4% of women were generally satisfied with communication of midwives and physicians during their labor and delivery N = 1196	*Reported* prevalence is *58.4%* of women were generally satisfied with communication of midwives and physicians during their labor and delivery	24.4% to 74.7% of women were satisfied with the communication throughout the birthing process
	Satisfaction with health workers’ attitude [56]	Welcoming at admission prior to arrival to the ward (Yes 78.25%), If the medical team identified himself to the patient (Yes 23.75%), Badly handling of the patient "bad medical team behavior" (Yes 21.25%), Courtesy, full attention and helpfulness of the medical team towards patients (Always 41.5%, usually 48.5%, sometimes 10%, never 0) N = 400	Assume always and usually = Yes *Calculated* prevalence is *67.7%* of women were satisfied with health workers’ attitude	
	Satisfaction with health workers’ communication skills [56]	Explanation of the treatment plan for delivery to mothers (yes 61.25%), Encouragement to ask about plan of treatment (yes 26.8%), Encouragement to ask about discharge time (yes 11.25%), Tell mothers about fasting before operation (yes 94.2%), Give instruction of care before discharge (yes 92.5%) N = 400	*Calculated* prevalence is *57.2%* of women were satisfied with the health workers’ communication skills	
	“Mothers’ experience of care related to client-provider interaction” [57]	“The way doctor treated her” (Excellent/good 301, satisfactory 56, bad/very bad 78), “The way nurse treated her” (Excellent/good 349, satisfactory 39, bad/very bad 47), Privacy maintained (Yes 374 No 61), “Provider listened to her questions” (Yes 224 No 31 didn't ask 180), “Provider explained her health status” (Yes 310, No 125), “Mother informed about baby's condition” (Yes 178 No 257) N = 435	Assume excellent/ good = Yes *Calculated* prevalence is *66.5%* of women having positive experience of care related to client provider interaction	
	Satisfaction with “interpersonal aspect of care” [57]	“Privacy maintained during care” (Total satisfied 88.7%), “Encouragement at delivery” (Total satisfied 82%), “The way doctor treated them” (Total satisfied 69.2%), “The way nurses treated them” (Total satisfied 77.7%), “The way workers treated them” (Total satisfied 56.1%) N = 435	*Calculated* Prevalence is *74.7%* of women were satisfied with interpersonal aspect of care	
	Dissatisfaction with intrapartum care [54]	X = 22.40; SD +—4.06; range 12–31 Findings indicated that the majority of women were not satisfied with intrapartum care Prevalence 75.6% of women were dissatisfied with intrapartum care N = 320	*Calculated* prevalence is *24.4%* of women were satisfied with intrapartum care	
	Satisfaction with childbirth experience [55]	X = 111.6 SD + −15.5; range 57–153 Women were not satisfied with their childbirth experience	No prevalence	
	“Not talked to any health professional about how they felt about what happened during labor and birth” [54]	Numbers were not clear to calculate the prevalence	No prevalence	
	Women’s satisfaction [51]	X = 133.3; range 45–155 High level of satisfaction	No prevalence	
“Lack of supportive care”	“Presence of birth support persons” [53]	16% of women had birth support persons presented	*Reported* prevalence is *16%* of women had birth support persons presented	4.14% to 16% of women had supportive care throughout the birthing process
	“Presence of companion” [57]	4.14% of women has companion presented with them N = 435	*Reported* prevalence is *4.14%* of women has companion presented with them	
	Women’s satisfaction [51]	X = 133.3; range 45–155 High level of satisfaction	No prevalence	
“Loss of autonomy”	“Women’s authority during childbirth” [53]	36% of women had authority during childbirth in institutions N = 69	*Reported* prevalence is *36%* of women had authority during childbirth	35% to 36% of women had autonomy throughout the birthing process *These two studies measured the same outcome within the same study methodological characteristics, but resulted with different numbers, and since it was not possible to decide on which number to consider, both prevalence were considered for the analysis
	“Women’s perceived authority at birth” [52]	35% perceived own authority during childbirth in institutions N = 68	*Reported* prevalence is *35%* of women perceived own authority during childbirth in institutions	
	Women’s satisfaction [51]	X = 133.3; range 45–155 High level of satisfaction	No prevalence	
	“Women’s perception of control” [51]	X = 44.9; range 10–70 Average level of perceived control in labor	No prevalence	
	“Perceived control during childbirth experience” [55]	X = 81.8 SD + −9.4; range 58–107 Women perceived that they had little control over their childbirth experience	No prevalence	
	Satisfaction with childbirth experience [55]	X = 111.6 SD + −15.5; range 57–153 Women were not satisfied with their childbirth experience	No prevalence	
	“Own choice of birth attendance” [53]	72.5% of women had their own choice of birth attendance/location (there is no prevalence for own choice of birth attendance only) N = 69	No prevalence	
Typology No. Seven: “Health system conditions and constraints”				
**“**Lack of resources”	Privacy sensation during hospital stay [56]	42.7% were least satisfied with privacy sensation during hospital stay N = 400	*Reported* prevalence is *57.3%* of women were satisfied with privacy sensation during hospital stay	55.5% to 89% of women were satisfied with the resources throughout the birthing process
	Maternal satisfaction related to facilities available in hospital [56]	•Breadth of the patient's or labor wardroom (Excellent 21.5%, very good 45%, good 0, suitable 33.5%, poor 0), Quietness in the patient's room (Excellent 0, very good 41.5%, good 55.5%, suitable 3%, poor 0), Cleanliness of the patient's room (Excellent 0, very good 31.25%, good 58%, suitable 10.75%, poor 0), Hand hygiene of the medical team (Excellent 0, very good 28.75%, good 50.75%, suitable 20.5%, poor 0), Bathroom facilities and cleanliness (Excellent 3.2%, very good 0, good 57%, suitable 39.8%, poor 0), Quality of food (No food 7%, excellent 0, very good 6.5%, good 38.25%, suitable 48.25%, poor 0) N = 400	Assume excellent, very good and good = Yes *Calculated* prevalence is *72.8%* of women were satisfied regarding facilities available in hospital	
	Satisfaction with “technical aspect of care” [57]	“Availability of medical facilities” (Total satisfied 62.9%), “Competency of care provider” (Total satisfied 92%), Health advice (Total satisfied 11.7%) N = 435	*Calculated* prevalence is *55.5%* of women were satisfied with technical aspect of care	
	“Satisfaction with physical environment” [57]	Cleanliness (Total satisfied 93.6%), Availability of beds (Total satisfied 95.2%), Sanitary facilities (Total satisfied 78.2%) N = 435	*Calculated* prevalence is *89%* of women were satisfied with physical environment	
	Satisfaction with “outcome of care” [57]	“Health condition of mothers” (Total satisfied 86.4%), “Health condition of the newborn” (Total satisfied 90.3%) N = 435	*Calculated* prevalence is *88.4%* of women were satisfied with outcome of care	
	Postpartum care [50]	81.7% in 2005 and 89.8% in 2008 of women received postpartum care after delivery in the hospital N = 9845 in 2005 and N = 7896 in 2008	*Reported* prevalence is *85.75%* of women received postpartum care after delivery in the hospital	
	“Mothers’ overall satisfaction with delivery care” [57]	63% of women were satisfied with the quality of delivery care they received at the hospital N = 435	*Reported* prevalence is *63%* of women were satisfied with the quality of delivery care they received at the hospital	
	“General satisfaction with care during labor” [47]	78% of women were generally satisfied with care during labor N = 1196	*Reported* prevalence is *78%* of women were generally satisfied with care during labor	
	Women’s satisfaction with intrapartum care [48]	Most women (98.7%) reported that they were satisfied with the care they received, but without distinguishing between women who gave birth in a health facility (78.8%) and women who gave birth at home (20.4%)	No prevalence	
	Satisfaction with childbirth experience [55]	X = 111.6 SD + −15.5; range 57–153 Women were not satisfied with their childbirth experience	No prevalence	
	Attendance of anyone that they didn’t want to be there [54]	Numbers were not clear to calculate the prevalence	No prevalence	
	Women’s satisfaction [51]	X = 133.3; range 45–155 High level of satisfaction	No prevalence	
“Lack of policies”	Mother-infant proximity [53]	“Skin-to-skin” (0), “Wrapped/dressed in arms” (15%, 10/66), “Separate bed/elsewhere near” (39.4%, 26/66), “Other place in room but out of contact” (36.4%, 24/66), “Separate room/place out of sight” (9%, 6/66) N = 66	Assume “skin-to-skin”, “wrapped/ dressed in arms”, “separate bed/ elsewhere near” = Yes *Calculated* prevalence is *54.4%* of women had close contact with their newborn after delivery	54.4% to 78.5% of women were satisfied with the policies throughout the birthing process
	Satisfaction with the admission process [56]	73.5% were satisfied with the admission process N = 400	*Reported* prevalence is *73.5%* of women were satisfied with the admission process	
	General assessment of the childbirth process [56]	Excellent 0, very good 23%, good 55.5%, suitable 16.25%, poor 5.25% N = 400	Assume excellent, very good and good = Yes Calculated prevalence is *78.5%* of women were satisfied with the childbirth process	
	Satisfaction with childbirth experience [55]	X = 111.6 SD + −15.5; range 57–153 Women were not satisfied with their childbirth experience	No prevalence	
	Women’s satisfaction [51]	X = 133.3; range 45–155 High level of satisfaction	No prevalence	
Typology No. Five: “Failure to meet professional standards”				
Physical examinations and procedures	Satisfaction with childbirth experience [55]	X = 111.6 SD + −15.5; range 57–153 Women were not satisfied with their childbirth experience	No prevalence	

### Findings for estimating the prevalence of mistreatment

This paper aimed to present prevalence estimates of mistreatment of women throughout the birthing process in health facilities in Arab countries. However, this was not possible due to high heterogeneity in the 11 studies. Therefore, the prevalence for each outcome of interest within each sub-category (either reported or calculated) was used to inform the overall prevalence range for each sub-category, as shown in Table 2.

The overall prevalence of women satisfied with the communication throughout the birthing process ranged between 24.4% and 74.7%. The overall prevalence of women who had supportive care at any point throughout the birthing process ranged between 4.14% and 16%. The overall prevalence of autonomy in women throughout the birthing process ranged between 35 and 36%. The overall prevalence of women who were satisfied with resources of the health system birthing process ranged between 55.5% and 89%. Finally, the overall prevalence of women who were satisfied with the health system's policies throughout the birthing process ranged between 54.4% and 78.5%.

### Risk bias assessment

The findings mentioned above were all based on the risk of bias judgments that are shown in Table 3. Six out of the 11 studies have a low overall risk of bias, while the remaining five showed a moderate overall risk of bias. The majority of the studies were judged to be high risk for the following items on the Hoy checklist [42]: bias for the target population, the sampling frame, and the random selection. As well as all studies were judged to be low risk of bias on the checklist for the case definition, the standard mode of data collection (i.e. data were collected from all the subjects using same mode), and the numerators and denominators (i.e. appropriate calculations for the parameters of interest).

**Table 3:** Risk bias assessment as per Hoy's criteria

First author/year	Target population	Sampling frame	Random selection	Non-response bias	Direct or proxy data collection	Case definition	Reliability and validity	Standard mode of data collection	Prevalence period	Numerators and denominators	Summary overall risk
Ahmed, 2020 [43]											
Bashour, 2005 [44]											
Boukhalfa, 2016 [45]											
Fort, 2012 [46]											
Kabakian-Khasholian, 2017 [47]											
Kempe, 2010 [48]											
Kempe, 2011 [49]											
Mohamma, 2014 [50]											
Monazea, 2015 [53]											
Oweis, 2009 [51]											
Sayed, 2018 (52)											

Red  = High risk,

Green  = Low risk,

Orange  = Moderate risk

## Discussion

### Estimating the prevalence of mistreatment of women

It was difficult to estimate the prevalence of mistreatment of women throughout the birthing process in health facilities in Arab countries because the tools and definitions were not consistent. Consequently, this review reported a broad range of prevalence for each sub-category. A similar finding was reported by Sando and colleagues’ systematic review, which demonstrated that the lack of standardized definitions, instruments, and study methods used in measuring disrespect and abuse affected the comparability between results and introduced systemic errors in reported prevalence estimates [16].

Moreover, using a single methodology and definition in the WHO study to estimate the prevalence of violence against women across ten countries and cultures significantly reduced the challenges faced by earlier conducted research. It also enabled comparability with the newly completed international research initiatives using the same methodology and definition. This resulted in gaining a more comprehensive picture of violence against women worldwide [54].

### The terminology used in measuring mistreatment of women

The terms used in the identified studies were not synonyms for the mistreatment of women. However, they were considered a proxy for mistreatment since they did not directly measure it. The terms found were more related to childbirth experience, the attitude of health personnel, and physician–patient relations. General terms such as “obstetric violence” and “disrespect” were not found in quantitative studies in Arab countries. A qualitative study conducted in 2017 used the terms “disrespect and abuse” and “mistreatment.” [55]. This indicates that such terms are newly introduced in Arab countries and are still being explored. However, they have yet to develop tools to measure them.

### The tools used in measuring mistreatment of women

The tools used to measure mistreatment varied widely in terms of the content, length, purpose, validation, and outcomes. This resulted in incomparable results between the included studies. As well as, indicating the essential need for utilizing a standardized tool in quantitative studies that are adequately adapted and validated. This finding agrees with the result of a systematic review that emphasized the necessity of providing a standard tool to accurately estimate the prevalence of women’s childbirth experiences and enable comparability [56].

Several studies that measured disrespect and abuse based on Browser and Hill categories employed different definitions of a standardized operational definition. This resulted in differences in prevalence estimates, thus facing the same problem of having incomparable results and preventing the possibility of conducting a meta-analysis [5, 57, 58]. This finding agrees with Nilver’s systematic review that even when researchers and clinicians use different instruments to measure the same construct of interest, it will be difficult to compare and statistically report the results of systematic reviews [59]. This emphasized that standardized terminology should be accompanied by the standardized tool.

### The typology used in measuring mistreatment of women

The types of mistreatment found did not exceed two main typologies out of the seven from the evidence-based typology- which are “poor rapport between women and providers” and “health system conditions and constraints.” It was apparent that the types used were found to be marginal in comparison with the ones mentioned in the evidence-based typology that the WHO developed. A theory behind why other types were not used was that researchers in Arab countries did not consider the remaining typologies in their research about childbirth experience as a priority to improve the quality of care. The result of not considering other types yielded tools that were not comprehensive enough to include additional domains of mistreatment or measure childbirth experience.

Other typologies were found in the qualitative studies [55, 60–64]. These studies included additional types such as: “physical abuse” [55, 61], “verbal abuse” [55, 60, 61, 60–61], “stigma and discrimination” [55, 60], and “failure to meet professional standards of care” (i.e., “neglect and abandonment” [55, 61, 63, 64], “lack of informed consent process” [62], and “physical examinations and procedures”) [62].

It seems that when women were allowed to speak their minds about their childbirth experience, they reflected on these four main typologies due to the negative impact it had on their childbirth experience [23]. Consequently, these typologies should be explicitly explored in future quantitative research in Arab countries. Also, the tools used in the quantitative studies should be comprehensive for all the typologies mentioned in the evidence-based typology. They should as well cover all dimensions of the childbirth experience.

Moreover, it was hard to find the typology of “sexual abuse” in health facilities in studies conducted in Arab countries because obtaining information about “sexual abuse” or “sexual violence” in Arab societies is not easy. Talking about sex, in general, is a sensitive topic in the Arab world, and cultural values reinforce silence and inaction around such events [65, 66].

### Methodological issues within the included studies

The timing for interviewing women to ask about mistreatment varied between studies as directly after the following childbirth or later up to a year after delivery. The timing of the interview is crucial and essential. The literature suggested that recall is more accurate in the postpartum period than immediately following childbirth when women are physically exhausted and have no time to mentally process the events that occurred during childbirth [16, 67]. Additionally, exit surveys on the facility's grounds may induce courtesy bias or reluctance to disappoint researchers by reporting negative experiences, especially when the interviewers were perceived to be affiliated with the same facility [5, 6]. Women may fear their opinions affecting their future use of services at the same facility, even if conducted privately [6, 68]. After a long time after birth, interviewing might over or underestimate the prevalence due to induced recall bias [8].

There were no published articles before the year 2005. Women’s experience during facility-based childbirth is a new and neglected area of research in Arab countries [69]. Since then, several studies in Egypt, Lebanon, Syria, and occupied Palestinian territory have been conducted to understand the experience of facility-based childbirth from a women’s point of view [69, 70].

### Strengths and methodological considerations of the current review

This systematic review was the first to be conducted among Arab countries. It showed the contribution of Arab countries to the research of mistreatment of women during facility-based childbirth. It also showed that this topic is still new and has not been tackled quantitatively yet. It is recommended that future researchers increase their research in this area using standardized definitions, tools, and study methods to accurately measure the mistreatment of women to enable comparison across time and between countries. It also prepares future researchers to face some challenges when using the WHO standardized tool because some terms and typologies have not been used in Arabic countries yet.

The hand searching yielded 17 new articles after excluding all reports, qualitative and randomized control trial designs, and doctorate dissertations. Moreover, the purpose behind the hand searching was to ensure that no relevant articles were missed during the search. In this paper, grey literature and unpublished reports were not screened because the focus was on published articles only.

This current review focused on the article's content rather than the quality of the research published to be inclusive.

## Recommendations

This review recommends that future quantitative studies in Arab countries use standardized and comprehensive terminology for the mistreatment of women. Also, a standardized tool that covers all aspects of mistreatment. In addition to a standardized timing, data collection method, data collection setting, and inclusion criteria to enhance comparability between results and allow pooling when estimating the prevalence. Still, this tool needs to be validated and adapted to suit Arab culture and context.

It is also recommended to update this review specifically to include qualitative studies related to mistreatment of women in Arab countries to provide sufficient information for a more holistic understanding of the mistreatment of women and further investigate what other terms and types would result.

Finally, it is recommended to use the evidence-based typology developed by WHO researchers since it has a clear definition for the mistreatment of women during facility-based childbirth and a standardized tool and methodology.

## Conclusion

The quantitative studies in Arab countries did not directly tackle the mistreatment of women throughout the birthing process in health facilities, and the resulting terms were a proxy for the word mistreatment. However, estimating the prevalence for these proxy terms was hard to obtain due to the heterogeneity of the terms used, tools used to operationalize the terms, inclusion/exclusion criteria, and the methodological characteristics of the conducted studies.

## Supplementary Information

**Additional file 1:** Search Keywords.

**Additional file 2:** List of full text excluded with reasons.

## Data Availability

All data generated or analyzed during this study are included in this published article.

## Abbreviation

WHO: World Health Organization
