# Supplementary material for: Women’s experiences throughout the birthing process in health facilities in Arab countries: a systematic review
Source: Reprod Health. 2022 Mar 18;19:68. doi: 10.1186/s12978-022-01377-y (PMC8931971; doi:10.1186/s12978-022-01377-y)
Supplement: Supplementary file 1 — Additional file 1: Search Keywords. [file 12978_2022_1377_MOESM1_ESM.docx]

| Field | Search Keywords |
| --- | --- |
| Keywords for mistreatment | 1. Mistreatment 2. Disrespect* 3. Abus* 4. Respect* 5. Neglect* 6. Confidentiality (MESH) 7. Informed consent 8. Physical abuse (MESH) 9. Dignity 10. Stigma 11. Assault 12. Attitude of health personnel (MESH) 13. Healthcare disparities (MESH) 14. Obstetric violence 15. Accessibility of health services (MESH) 16. Birth experience 17. Childbirth experience 18. Labor experience 19. Labour experience 20. Physician-Patient Relations (MESH) |
| Keywords for health facilities | 1. Health facilities (MESH) 2. Delivery rooms (MESH) 3. Facility-based childbirth 4. Birthing centers (MESH) 5. Obstetrics and Gynecology Department, Hospital (MESH) 6. Nursing Service, Hospital (MESH) 7. Maternal-Child Health Centers (MESH) 8. Ambulatory health center (MESH) 9. Maternity hospitals (MESH) 10. “Institutional childbirth” 11. “Institutional delivery” |
| Keywords for childbirth | 1. Parturition (MESH) 2. Delivery, obstetric (MESH) 3. Labor, obstetric (MESH) 4. “Obstetric care” 5. Postnatal Care (MESH) 6. Perinatal care (MESH) 7. Maternal health services (MESH) 8. Maternal health (MESH) 9. Maternal-Child Nursing (MESH) 10. Women’s health services (MESH) 11. Obstetric nursing (MESH) 12. Prenatal care (MESH) 13. Intrapartum 14. Intra-partum 15. Postpartum 16. Post-partum 17. Intranatal |
| Keywords for Arab countries | 1. Arabs (MESH) 2. Middle East (MESH) 3. Jordan 4. Palestine 5. Palestinian Authority 6. State of Palestine 7. Syria 8. Lebanon (MESH) 9. Morocco (MESH) 10. Mauritania 11. Algeria 12. Tunisia 13. Libya 14. Sudan (MESH) 15. Somalia 16. Egypt (MESH) 17. Saudi Arabia (MESH) 18. Yemen (MESH) 19. Oman (MESH) 20. Qatar (MESH) 21. Bahrain, 22. Kuwait, 23. Comoros (MESH) 24. Iraq (MESH) 25. Djibouti (MESH) 26. United Arab Emirates (MESH) |
