# Supplementary material for: Women’s experiences throughout the birthing process in health facilities in Arab countries: a systematic review
Source: Reprod Health. 2022 Mar 18;19:68. doi: 10.1186/s12978-022-01377-y (PMC8931971; doi:10.1186/s12978-022-01377-y)
Supplement: Supplementary file 2 — Additional file 2: List of full text excluded with reasons. [file 12978_2022_1377_MOESM2_ESM.docx]

# Additional File 3: List of Full Text Excluded with Reasons

| First Author, year | Study Title | Reason for exclusion |
| --- | --- | --- |
| Abushaikha, 2007 (1) | “Methods of coping with labor pain used by Jordanian women” | Not cross-sectional, cohort or descriptive study designs |
| Ahamadani, 2014 (2) | “Perinatal health care in a conflict-affected setting: evaluation of health-care services and newborn outcomes at a regional medical centre in Iraq” | Not cross-sectional, cohort or descriptive study designs |
| Al-Rukeimi, 2017 (3) | “High rate of uterine rupture in a conflict setting of Hajjah, Yemen” | Not related to mistreatment of women during childbirth |
| AlSerouri, 2009 (4) | “Reducing maternal mortality in Yemen: challenges and lessons learned from baseline assessment” | Not related to mistreatment of women during childbirth |
| Arafa, 2000 (5) | “Outcomes of pregnancies complicated by early vaginal bleeding” | Not related to mistreatment of women during childbirth |
| Carlson, 2011(6) | “Fifty years of Sudanese hospital-based obstetric outcomes and an international partnership” | Abstracts only, reports and conferences |
| Couillet, 2007 (7) | “The use of antenatal services in health centres of Fès, Morocco” | Not related to mistreatment of women during childbirth |
| Dhaher, 2008 (8) | “Factors associated with lack of postnatal care among Palestinian women: a cross-sectional study of three clinics in the West Bank” | Not related to mistreatment of women during childbirth |
| Fouly, 2018 (9) | “Audit for quality of care and fate of maternal critical cases at Women's Health Hospital” | Not related to mistreatment of women during childbirth |
| Giacaman, 2007 (10) | “The limitations on choice: Palestinian women's childbirth location, dissatisfaction with the place of birth and determinants” | Not related to mistreatment of women during childbirth |
| Gray, 2019 (11) | “Obstetric violence: Clinical staff perceptions from a video of simulated practice” | Not related to mistreatment of women during childbirth |
| Handelzalts, 2016 (12) | “The association of birth model with resilience variables and birth experience: Home versus hospital birth” | Not Arab country |
| Hatamleh, 2013 (13) | “Evaluating the experience of Jordanian women with maternity care services” | Not cross-sectional, cohort or descriptive study designs |
| Kempe, 2011  (14) | “Veiled powersof culture: Autonomy and choice among childbearing women in the Arab world” | Abstracts only, reports and conferences |
| Mizrachi, 2017 (15) | “Does midwife experience affect the rate of severe perineal tears?” | Not Arab country |
| Sweidan, 2008 (16) | “Hospital policies and practices concerning normal childbirth in Jordan” | Not related to mistreatment of women during childbirth |
| Tappis, 2017 (17) | “Maternal Health Care Utilization Among Syrian Refugees in Lebanon and Jordan” | Not related to mistreatment of women during childbirth |
| VanLerberghe, 2014 (18) | “Country experience with strengthening of health systems and deployment of midwives in countries with high maternal mortality” | Not related to mistreatment of women during childbirth |
| Vogel, 2014 (19) | “Maternal complications and perinatal mortality: findings of the World Health Organization Multicountry Survey on Maternal and Newborn Health” | Not related to mistreatment of women during childbirth |
| Wick, 2005 (20) | “Childbirth in Palestine” | Not related to mistreatment of women during childbirth |
| Zimmo, 2018 (21) | “Episiotomy practice in six Palestinian hospitals: a population-based cohort study among singleton vaginal births” | Not related to mistreatment of women during childbirth |

1. Abushaikha LA. Methods of coping with labor pain used by Jordanian women. Journal of Transcultural Nursing. 2007;18(1):35-40.

2. Ahamadani F, Louis H, Ugwi P, Hines R, Pomerleau M, Ahn R, et al. Perinatal health care in a conflict-affected setting: evaluation of health-care services and newborn outcomes at a regional medical centre in Iraq. EMHJ-Eastern Mediterranean Health Journal. 2014;20(12):789-95.

3. Al-Rukeimi AA, Al-Haddad A, Ali AA, Adam I. High rate of uterine rupture in a conflict setting of Hajjah, Yemen. Journal of Obstetrics and Gynaecology. 2017;37(8):1106-7.

4. Al Serouri AW, Al Rabee A, Bin Afif M, Al Rukeimi A. Reducing maternal mortality in Yemen: challenges and lessons learned from baseline assessment. International Journal of Gynecology & Obstetrics. 2009;105(1):86-91.

5. Arafa M. Outcomes of pregnancies complicated by early vaginal bleeding. EMHJ-Eastern Mediterranean Health Journal, 6 (2-3), 457-464, 2000. 2000.

6. Carlson V, Omer M, Ibrahim S, Ahmed S, O’Byrne K, Kenny L, et al. Fifty years of Sudanese hospital‐based obstetric outcomes and an international partnership. BJOG: An International Journal of Obstetrics & Gynaecology. 2011;118(13):1608-16.

7. Couillet M, Serhier Z, Tachfouti N, Elrhazi K, Nejjari C, Perez F. The use of antenatal services in health centres of Fes, Morocco. Journal of Obstetrics and Gynaecology. 2007;27(7):688-94.

8. Dhaher E, Mikolajczyk RT, Maxwell AE, Krämer A. Factors associated with lack of postnatal care among Palestinian women: A cross-sectional study of three clinics in the West Bank. BMC Pregnancy and Childbirth. 2008;8(1):26.

9. Fouly H, Abdou FA, Abbas AM, Omar AM. Audit for quality of care and fate of maternal critical cases at Women's Health Hospital. Applied Nursing Research. 2018;39:175-81.

10. Giacaman R, Abu-Rmeileh NM, Wick L. The limitations on choice: Palestinian women's childbirth location, dissatisfaction with the place of birth and determinants. The European Journal of Public Health. 2007;17(1):86-91.

11. Gray T, Mohan S, Lindow S, Farrell T. Obstetric violence: Clinical staff perceptions from a video of simulated practice. European Journal of Obstetrics & Gynecology and Reproductive Biology: X. 2019;1:100007.

12. Handelzalts JE, Zacks A, Levy S. The association of birth model with resilience variables and birth experience: Home versus hospital birth. Midwifery. 2016;36:80-5.

13. Hatamleh R, Shaban IA, Homer C. Evaluating the experience of Jordanian women with maternity care services. Health Care for Women International. 2013;34(6):499-512.

14. Kempe A. Veiled power sof culture: Autonomy and choice among childbearing women in the Arab world. Journal of Obstetrics and Gynaecology. 2011;31(1):17.

15. Mizrachi Y, Leytes S, Levy M, Hiaev Z, Ginath S, Bar J, et al. Does midwife experience affect the rate of severe perineal tears? Birth. 2017;44(2):161-6.

16. Sweidan M, Mahfoud Z, DeJong J. Hospital policies and practices concerning normal childbirth in Jordan. Studies in Family Planning. 2008;39(1):59-68.

17. Tappis H, Lyles E, Burton A, Doocy S, Team JHAS, Team LHAS. Maternal health care utilization among Syrian refugees in Lebanon and Jordan. Maternal and Child Health Journal. 2017;21(9):1798-807.

18. Van Lerberghe W, Matthews Z, Achadi E, Ancona C, Campbell J, Channon A, et al. Country experience with strengthening of health systems and deployment of midwives in countries with high maternal mortality. The Lancet. 2014;384(9949):1215-25.

19. Vogel J, Souza J, Mori R, Morisaki N, Lumbiganon P, Laopaiboon M, et al. Maternal complications and perinatal mortality: findings of the World Health Organization Multicountry Survey on Maternal and Newborn Health. BJOG: An International Journal of Obstetrics & Gynaecology. 2014;121:76-88.

20. Wick L, Mikki N, Giacaman R, Abdul-Rahim H. Childbirth in palestine. International Journal of Gynecology & Obstetrics. 2005;89(2):174-8.

21. Zimmo K, Laine K, Fosse E, Zimmo M, Ali-Masri H, Zucknick M, et al. Episiotomy practice in six Palestinian hospitals: a population-based cohort study among singleton vaginal births. BMJ Open. 2018;8(7):e021629.
